# Engineering a stable high-copy episomal plasmid in *Yarrowia lipolytica*

**DOI:** 10.1093/femsyr/foag054

**Published:** 2026-10-13

**Authors:** Fengyi Li, Sirui Peng, Huangwei Xu, Yalin Guo, Qingsheng Qi, Jin Hou

**Affiliations:** Shandong University, State Key Laboratory of Microbial Technology, Binhai Road 72, Qingdao 266237, P. R. China; Shandong University, State Key Laboratory of Microbial Technology, Binhai Road 72, Qingdao 266237, P. R. China; Shandong University, State Key Laboratory of Microbial Technology, Binhai Road 72, Qingdao 266237, P. R. China; Shandong University, State Key Laboratory of Microbial Technology, Binhai Road 72, Qingdao 266237, P. R. China; Shandong University, State Key Laboratory of Microbial Technology, Binhai Road 72, Qingdao 266237, P. R. China; Shandong University, State Key Laboratory of Microbial Technology, Binhai Road 72, Qingdao 266237, P. R. China

**Keywords:** *Yarrowia lipolytica*, episomal plasmids, ARS1, ubiquitin-mediated protein degradation, plasmid copy number, synthetic biology

## Abstract

Episomal plasmids are widely used in yeast metabolic engineering for their flexibility and high transformation efficiency. However, their application in *Yarrowia lipolytica* is often limited by low copy number and insufficient genetic stability. In this study, a robust episomal plasmid that balanced genetic stability and protein expression capacity was developed in *Y. lipolytica*. The autonomously replicating sequence (ARS) elements were selected, and ARS1 was identified as a favorable element that balanced genetic stability and gene expression capacity. To improve plasmid copy number, different ubiquitin degrons were used to tune selection marker abundance, and Ubi-R-Δk-mediated marker resulted in an approximately fourfold increase in plasmid copy number (from ∼1 to ∼5 copies per cell). The copy number of the engineered plasmid was one of the highest–copy episomal systems in *Y. lipolytica*. To further evaluate the engineered plasmid, we applied it to β–carotene biosynthesis. The results showed that the β–carotene titer was 3.43–fold higher than that of the non–optimized plasmid. Collectively, this work expands the episomal plasmid toolbox for *Y. lipolytica* and establishes a generalizable framework for robust protein expression plasmid engineering in non-conventional yeasts.

## Teaser

A stable high-copy plasmid system was developed for Yarrowia lipolytica, enabling stronger gene expression and improved production of valuable compounds.

## Introduction

Plasmids are widely employed in basic research and synthetic biology owing to their ease of use, host transfer flexibility, and high transformation efficiency (Dolan et al. 2022, Martínez-García et al. 2023). These features make them particularly suitable for library construction and high-throughput screening. *Yarrowia lipolytica* has emerged as a prominent non-conventional yeast for its characteristics of high robustness, broad substrate utilization spectrum, and strong lipid metabolism (Jagtap et al. 2021, Kobus et al. 2025). These inherent traits have enabled its widespread application in the biosynthesis of lipids, organic acids, terpenoids, and other value-added compounds (Liu et al. 2014, Park et al. 2023). However, unlike the model organism *Saccharomyces cerevisiae*, which has both high-copy 2-μm plasmids and low-copy plasmids based on centromere (CEN)/autonomously replicating sequences (ARS), *Y. lipolytica* only has CEN/ARS-based plasmids. ARS serves as the binding site for the origin recognition complex, thereby initiating plasmid DNA replication. CEN is involved in plasmid partitioning by interacting with centromere-binding proteins, ensuring that the replicated plasmids are accurately segregated into two daughter cells during cell division (Vernis et al. 2001). Consequently, these plasmids are regulated by the centromere-mediated segregation and are maintained at a low copy number. These plasmids are typically maintained at 1–3 copies per cell (Bredeweg et al. 2017, Dulermo et al. 2017). Due to their low copy number and limited genetic stability, these vectors are generally unsuitable for stable, high-level heterologous protein expression.

Episomal CEN/ARS-based plasmids in *Y. lipolytica* have been optimized through engineering ARS/CEN elements, exploiting mitochondrial DNA-derived replication origins (mtORI), or modulating the effective dosage of selection markers. For example, fusing a promoter upstream of the CEN region has been reported to increase the plasmid copy number by approximately 80% (Liu et al. 2014). Random mutagenesis of CEN sequences has been employed to improve plasmid stability (Li et al. 2026). More recently, mtORI-based plasmids have been reported to reduce the plasmid loss rate by 40%, and additional weakening of selection marker promoters has been shown to increase the copy number to 2–5 per cell (Cui et al. 2021). However, none of these strategies has yielded an effective episomal system that simultaneously achieves high genetic stability and high expression capacity in *Y. lipolytica*.

In this study, we engineered the CEN/ARS plasmid to construct a stable, high-expression plasmid platform in *Y. lipolytica*. ARS elements were screened and ARS1 was identified as the optimal element for balancing stability and expression. To improve the plasmid copy number, a ubiquitin–mediated degradation strategy was implemented to fine–tune selection marker abundance. The Ubi–R–Δk tag increased plasmid copy number approximately fourfold, achieving a copy number comparable to that of mtORI–based plasmids, which currently represent the highest–copy episomal system in *Y. lipolytica*. Importantly, the protein expression capacity of the engineered plasmid was superior to that of the mtORI-based plasmids. This study represents the first application of the Ubi–R–Δk tag for modulating plasmid copy number. This work expands the episomal plasmid toolbox for *Y. lipolytica* and provides a generalizable framework for engineering high–level protein expression plasmids in other non–conventional yeasts.

## Materials and methods

### Strains and culture conditions

*Escherichia coli* DH5α was used to construct the recombinant plasmids. *E. coli* was cultured in Luria − Bertani (LB) medium containing 5 g/L yeast extract, 10 g/L tryptone, and 10 g/L NaCl at 37°C for 12-16 h. Ampicillin (100 μg/mL) was added to the LB medium for selecting the positive transformants. *Y. lipolytica* strains were cultivated at 30°C in YPD medium (20 g/L glucose, 10 g/L yeast extract, and 20 g/L tryptone) or SC-Leu (6.7 g/L yeast nitrogen base, 2.0 g/L ammonium sulfate and 0.7 g/L complete supplement mixture lacking leucine). Yeast extract and tryptone were purchased from Oxoid (Basingstoke, England). Ampicillin was purchased from Solarbio (Beijing, China). Other chemicals, if not specified otherwise, were purchased from Sinopharm Chemical Reagent Co., Ltd. (Shanghai, China). *Y. lipolytica* Po1f and its derived strains were listed in Table S1.

### Plasmids and strains construction

The YLEP-leu-*hrGFP* plasmid was used as the backbone for plasmids construction. All plasmids were listed in Table S1 and primers were listed in Table S2. The genomic DNA of Po1f was used as a template for amplifying ARS1 and mtORI fragments. The genes coding phytoene synthase/lycopene cyclase (*CarRP*), phytoene dehydrogenase (*CarB*) and a series of Ubi-tags were codon-optimized for expression in *Y. lipolytica* (Beijing, China). All sequences of heterologous proteins were listed in Table S4. Polymerase chain reaction (PCR) was performed according to the manufacturer’s instructions for the DNA polymerase, Phanta Max Super-Fidelity DNA Polymerase (Jiangsu, China). The homologous sequence for DNA assembly was introduced using PCR primers. PCR products were ligated using 2 × MultiF Seamless Assembly Mix purchased from ABclonal Technology (Hubei, China). Then, the ligation products were introduced into *E. coli* DH5α.

### Transformation efficiency and fluorescence intensity

The transformation efficiency was calculated as follows. Fresh *Y. lipolytica* cells were transformed with 1.0 μg plasmid DNA using the lithium acetate method, and transformants were spread on selective plates. The resulting colonies were counted after cultivation for 2–3 days, and the transformation efficiency was calculated by dividing the number of surviving colonies on the selective plates by the DNA quantity.

To characterize the *hrGFP* expression of transformants, fluorescence intensity (excitation at 485 nm and emission at 528 nm), and cell growth (OD_600_) were detected by a MultiDetection Microplate Reader (Synergy HT, Biotek, USA).

### Plasmid stability measurement

The stability of the plasmid was assessed using a classical assay previously reported (Chatre et al. 2011). Strains harboring different episomal plasmids were pre-cultured in SC-Leu overnight at 30°C. Then, the initial culture was transferred into selective SC-Leu medium and rich YPD medium. The cultures were incubated at 30°C and 220 rpm for 96 h. Samples were taken every 24 h. After incubation, the culture was appropriately diluted and spread on selective and rich medium, and the obtained colonies were used to calculate the plasmid loss rate. Plasmid stability was evaluated based on the plasmid loss rate.

### Quantitative PCR (qPCR) for plasmid copy number determination

Plasmid copy number was determined via qPCR by comparing Cq values of the target gene (*GFP*) with single-copy endogenous reference gene (*GAPDH*, coding glyceraldehyde-3-phosphate dehydrogenase). To generate standard curves for both genes, standard plasmids T-*GAPDH* and T-*GFP* were constructed as follows. Fragments of *GAPDH* and *GFP* were amplified using *Y. lipolytica* genomic DNA and the YLEP-ORICEN-*hrGFP* plasmid as templates, respectively. The resulting PCR products were purified and individually ligated into the pMD19-T vector, yielding the standard plasmids T-*GAPDH* and T-*GFP*.

To generate standard curves, ten-fold serial dilutions of each standard plasmid ranging from 10³ to 10^7^ copies/μL were used as qPCR templates. Plasmid concentrations were measured and converted to copy numbers using the formula: copies/μL = [6.02 × 10²³ × (ng/μL × 10⁻⁹)]/(DNA length × 660) (Liu et al. 2025). Standard curves were generated by plotting the Cq values against the log₁₀ of the initial template copy number.

For copy number determination, total DNA extracted from *Y. lipolytica* transformants was used as the qPCR template. The Cq values of *GFP* and *GAPDH* were converted to the copy numbers according to their corresponding standard curves. The plasmid copy number was calculated as the ratio of *GFP* copy number to *GAPDH* copy number. qPCR was performed on an Applied Biosystems QuantStudio 3 Real-Time PCR System using SYBR Green Realtime PCR Master Mix (TOYOBO, Shanghai, China). The primers used for qPCR are listed in Table S3.

### β-carotene biosynthesis and quantification

The engineered *Y. lipolytica* Car0, Car1, Car2 and Car3 were cultured on SC-Leu-agar plates at 30°C for 3 days and then inoculated into test tubes containing 1.5 mL of SC-Leu medium at 30°C for 12 h with shaking at 220 rpm. Subsequently, yeast cells were transferred into 20 mL of SC-Leu medium in a 100 mL shake flask to synthesize β-carotene at OD_600_ = 1. The broth was cultured under the condition of 30°C and 220 rpm for 96 h.

β-carotene was quantified by collecting 20 μL of culture, extracting the pigments with 750 μL DMSO at 55°C for 10 min, adding an equal volume of acetone and incubating at 50°C for 10 min, followed by centrifugation at 12,000 rpm for 10 min. The supernatant was analyzed using an HPLC system (Shimadzu LC-20AT, Japan) equipped with a variable-wavelength detector and a Shim-pack GIST C18 column (5 µm, 4.6  ×  150 mm; Shimadzu). The mobile phases consisted of (A) methanol:acetonitrile:dichloromethane (42:42:16, v/v/v) and (B) acetonitrile:water (80:20, v/v), delivered at 1.0 mL/min and 30 °C. Commercial β-carotene (Sigma-Aldrich, Shanghai, China) was used as the standard.

## Results

### Screening autonomous replication origins to improve plasmid stability and protein expression level

ARS1 is one of the earliest well–characterized autonomously replicating sequences. The ARS1 in *Y. lipolytica* is typically composed of a replication origin (ORI), a centromere (CEN), and an intervening linker sequence (Vernis et al. 2001). Subsequent work demonstrated that the linker region is not essential for plasmid replication and segregation, leading to the development of a minimal ORI–CEN element. This ORI–CEN element is a truncated version of ARS1 lacking the linker region and has become a frequently used ARS in *Y. lipolytica* (Yamane et al. 2008). More recently, mitochondrial replication origins (mtORI) have been reported as plasmid elements conferring lower plasmid loss rates (Cui et al. 2021).

To evaluate the performance of different ARS elements, we constructed episomal plasmids harboring distinct ARS elements (ORI–CEN, ARS1, and mtORI) using the YLEP–ORICEN–*hrGFP* plasmid stored in our laboratory as the backbone. GFP served as a reporter to evaluate gene expression. After 36 h of cultivation, we compared the fluorescence intensity levels and copy numbers of different transformants. For transformants carrying the YLEP–ORICEN–*hrGFP* and YLEP–ARS1–*hrGFP* plasmids, the plasmid copy number were similar. The copy number of most transformants was between 0.8 and 1.5 (Figs. 1A and 1B). The YLEP–ARS1–*hrGFP* plasmid exhibited approximately 50% higher GFP fluorescence intensity than the YLEP–ORICEN–*hrGFP* plasmid. In contrast, transformants carrying the YLEP–mtORI–*hrGFP* plasmid exhibited a broader variation of fluorescence intensity and copy number (Fig. 1C). Among the eight transformants analyzed, the highest copy number was 2, and the corresponding transformant showed GFP fluorescence levels comparable to those carrying the YLEP–ARS1–*hrGFP* plasmid. However, most transformants maintained plasmid copy numbers between 1 and 1.5. GFP fluorescence intensity of the YLEP–mtORI–*hrGFP* plasmid was generally lower than that of the YLEP–ARS1–*hrGFP* plasmid.

**Figure 1:**
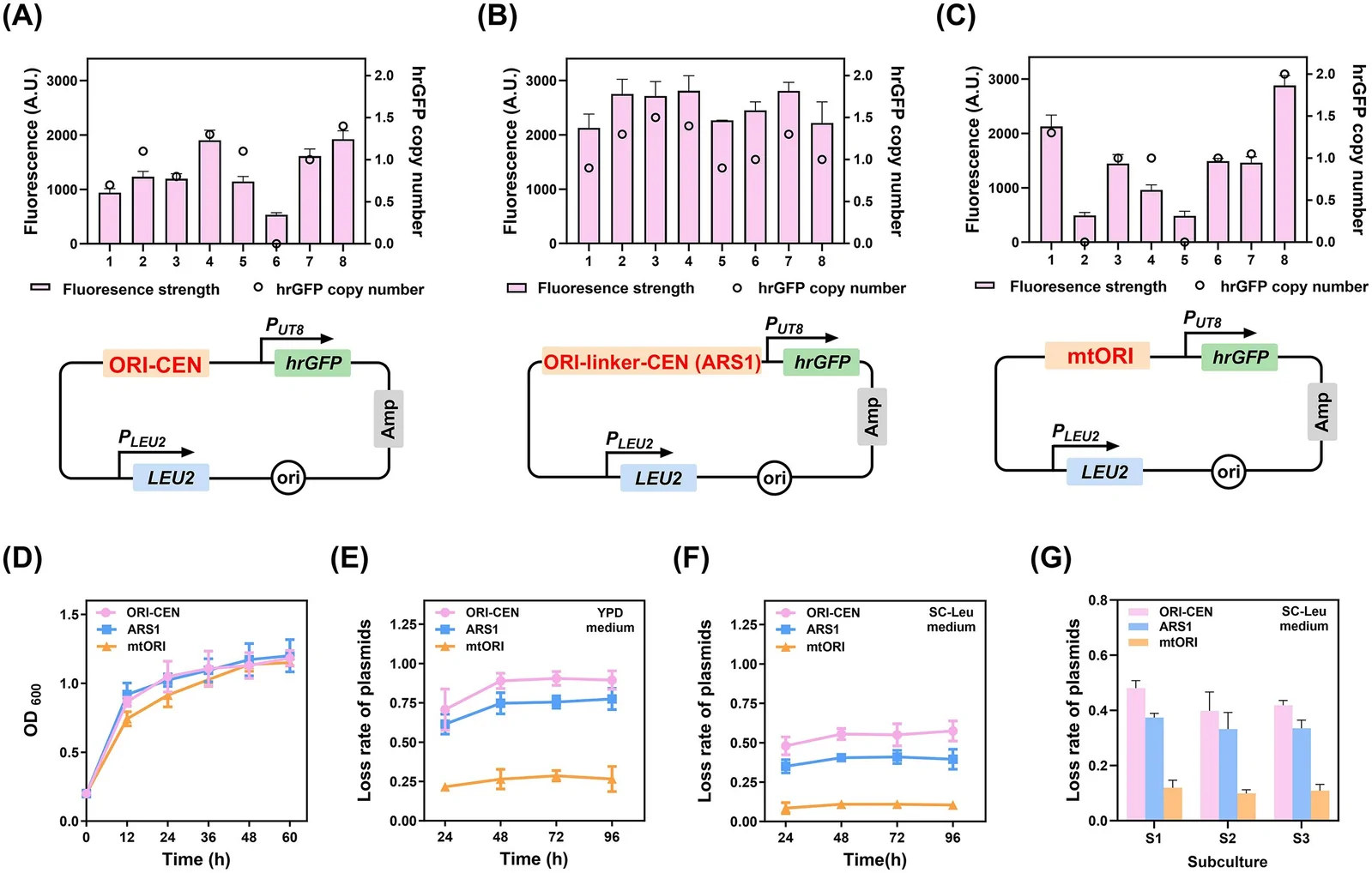
The selection of different ARS elements. The fluorescence intensity and plasmid copy number of transformants containing YLEP-ORICEN-*hrGFP* plasmids (A), YLEP-ARS1-*hrGFP* plasmids (B) and YLEP-mtORI-*hrGFP* plasmids (C). (D) The cell growth of transformants containing different plasmids. Determination of the loss rate of different plasmids in the rich (E) or selective medium (F). (G) Plasmid loss rates over three subcultures S1, S2 and S3 in selective medium. Data represent the mean ± SD of two or three biological replicates.

Transformation efficiency was subsequently evaluated for all three plasmids. As summarized in Table 1, the transformation efficiency of the ORI-CEN-based and ARS1-based plasmids (∼7.0 × 10² colonies μg⁻¹ DNA) was 184% higher than that of the mtORI-based plasmids (∼3.8 × 10² colonies μg⁻¹ DNA). The transformants containing different plasmids showed similar cell growth (Fig. 1D)

**Table 1:** The transformation efficiency of plasmids carrying different ARS elements.

Plasmids	Plasmid length	Colonies/μg DNA
YLEP-ORICEN-*hrGFP*	6702 bp	740
YLEP-ARS1-*hrGFP*	7612 bp	710
YLEP-mtORI-*hrGFP*	7129 bp	380

To further assess plasmid performance, plasmid loss rates were quantified under both selective (SC-Leu) and non–selective (YPD) conditions. Three independent transformants exhibiting the highest fluorescence intensities for each construct were selected for analysis. In non–selective YPD medium, loss rates stabilized after approximately 48 h of cultivation, reaching approximately 25%, 75%, and 89% for the mtORI–, ARS1–, and ORI–CEN–based plasmids, respectively (Fig. 1E). Under selective conditions, the corresponding loss rates were approximately 10.2%, 40.3%, and 55.1% (Fig. 1F). The plasmid copy numbers remained stable at the end of cultivation compared to the beginning (Fig. S1A).

To verify the robustness of the plasmid system for bioproduction, transformants carrying different plasmids were serially subcultured in selective SC–Leu medium for three subcultures S1, S2 and S3 (1:100 dilution every 48 h, ∼144 h total). As presented in Fig. 1G, plasmid loss rates remained relatively stable across subcultures for all three constructs, ranging from 9.8%–11.9%, 33.2%–37.4%, and 39.8%–48.1% for the mtORI–, ARS1–, and ORI–CEN–based plasmids, respectively. In addition, no significant changes in copy number were observed across the three subcultures (Fig. S1B). These results indicate that the mtORI–based plasmid exhibited better mitotic stability, followed by ARS1, while the ORI–CEN-based plasmid has the highest loss rate.

Taken together, although the mtORI-based plasmids exhibited better stability, the ARS1-based plasmids provided a more favorable balance between mitotic stability, transformation efficiency, and gene expression level. Therefore, the ARS1-based plasmids were selected for further optimization.

Although the stability and GFP expression level of plasmids were improved by screening ARS elements, the plasmid copy number remained relatively low. A previous study demonstrated that fusing different promoters upstream of the CEN region regulates centromere function, thereby enhancing plasmid copy number and gene expression (Liu et al. 2014). Among the tested promoters, the truncated TEF promoter (P_TEF(272)_) exhibited the most significant improvement (Liu et al. 2014). Therefore, the promoter P_TEF(272)_ was introduced upstream of the CEN region in the YLEP–ARS1–*hrGFP* plasmids. However, as shown in Fig. 2, this fusion did not increase fluorescence intensity, suggesting that protein expression levels were not significantly enhanced by promoter introduction. Thus, alternative strategies are required to increase plasmid copy number and gene expression level.

**Figure 2:**
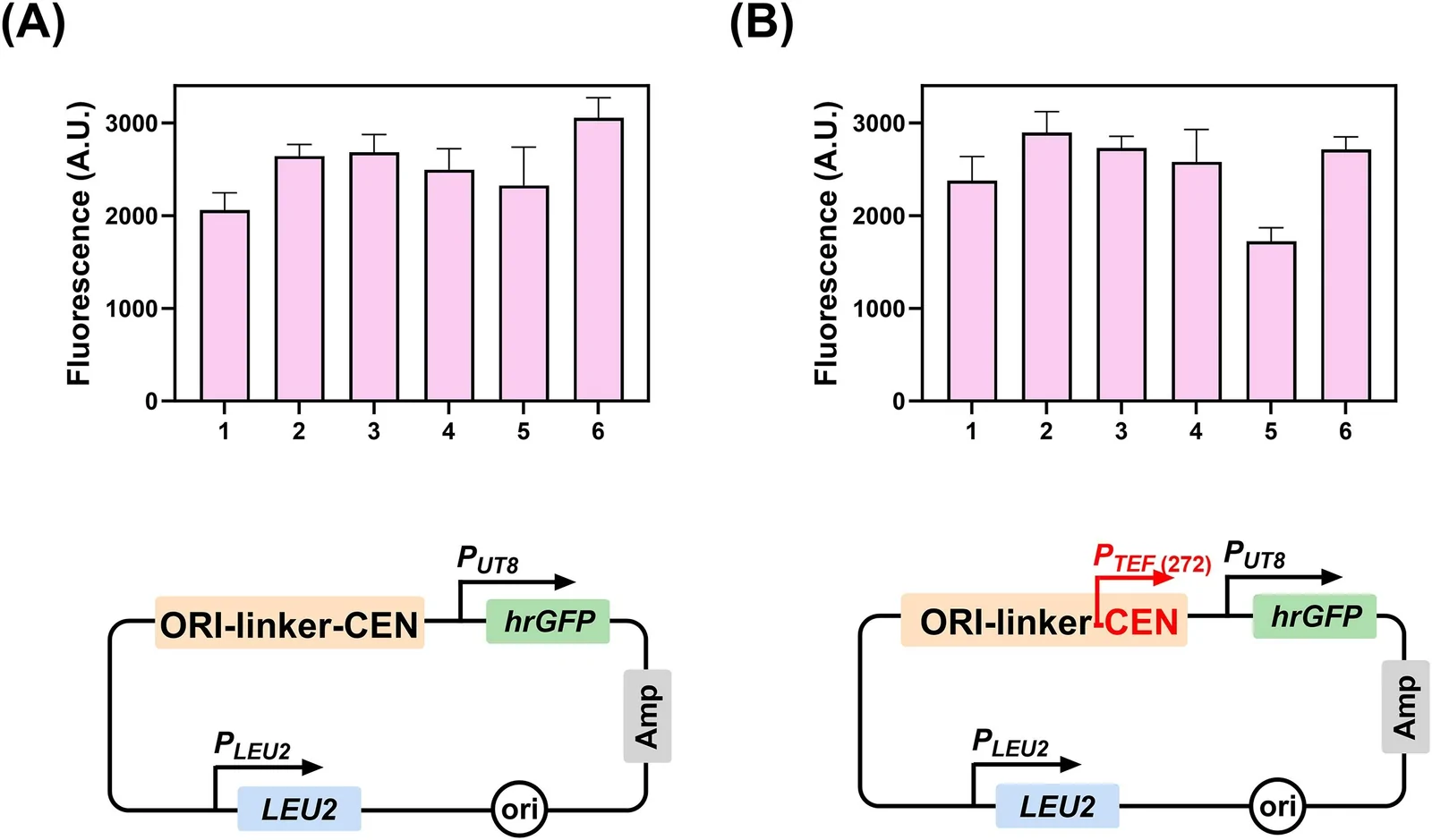
Introducing a promoter upstream of the CEN region does not affect the gene expression level. The fluorescence intensity of transformants containing YLEP-ARS1-*hrGFP* plasmids(A). and YLEP-ARS1-*hrGFP* plasmids with a promoter fused upstream of the CEN region(B). Data represent the mean ± SD of two or three biological replicates.

### Modulating selection marker abundance to enhance plasmid copy number

The expression level of selection marker is a critical determinant of plasmid copy number in yeast. Reducing the abundance of the marker protein intensifies the selection pressure, thereby favoring cells that maintain higher plasmid copy number to support growth under selective conditions (Li et al. 2023). As shown in Figs. 3A-C, the ubiquitin–proteasome system mediates selective protein degradation via exposure of destabilizing *N*-terminal residues following Ubi-tag in eukaryotic cells (Varshavsky 2019). These destabilizing amino acids mainly include arginine (R), lysine (K), leucine (L), tryptophan (W), and others. Arginine (R) is the most potent destabilizing *N*-terminal residue (Chen et al. 2012). In recent years, the Ubi-R tag was further engineered to increase degradation strength through the incorporation of the full-length (ek) or partially truncated (Δk) forms of an arginine- and lysine-rich KRK motif, resulting in the generation of the Ubi-R-ek and Ubi-R-Δk tags (Hackett et al. 2006, Houser et al. 2012). Accordingly, in this study, three Ubi-tags (Ubi-R, Ubi-R-Δk, and Ubi-R-ek) with distinct degradation strengths were designed to reduce the abundance of marker protein (Leu2), thereby improving plasmid copy number.

**Figure 3:**
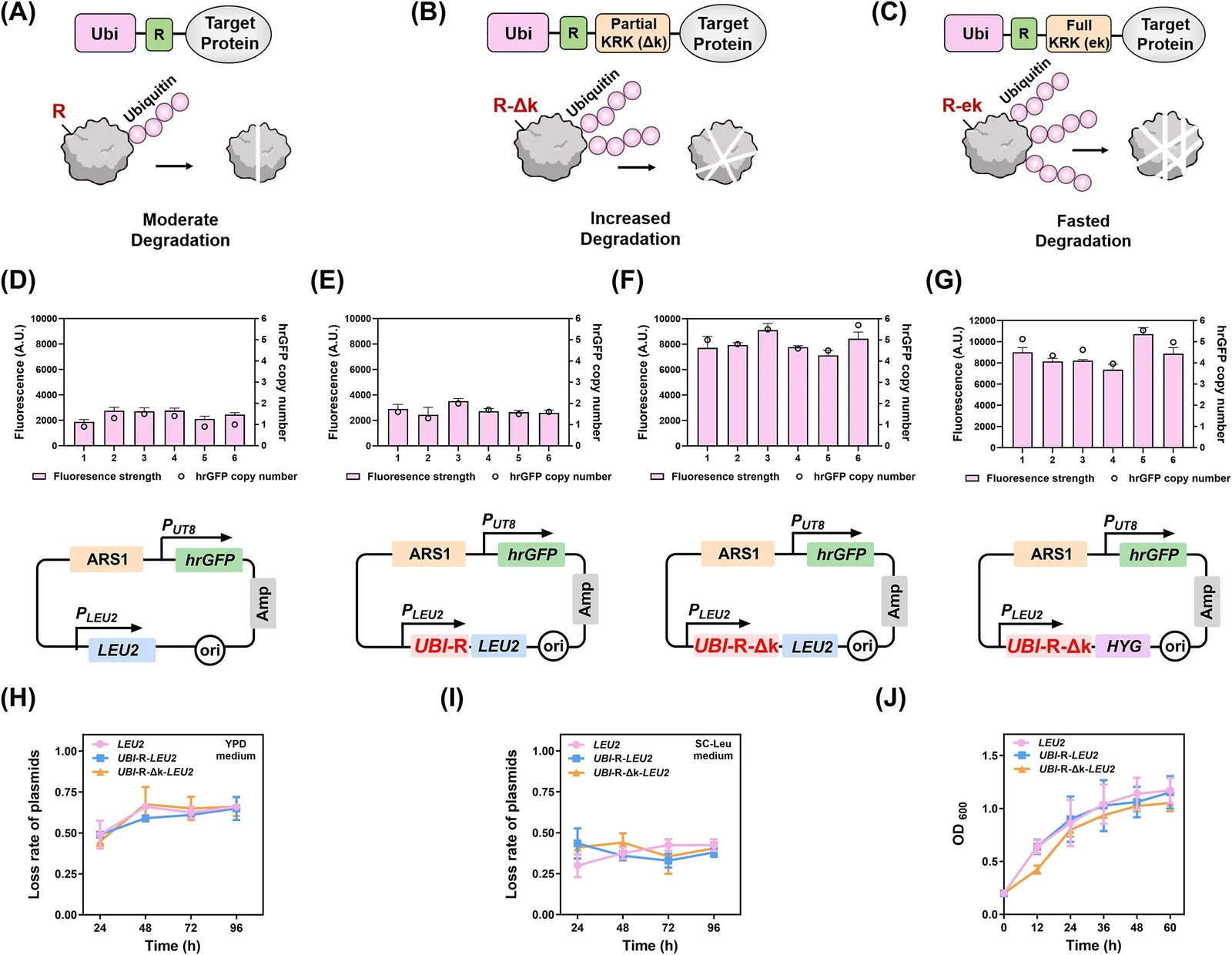
Modulating selection marker abundance for improved plasmid copy number. The schematic of the *N*-degron based protein degradation system based on (A) Ubi-R, (B) Ubi-R-Δk, and (C) Ubi-R-ek tags. The fluorescence intensity and copy number of transformants containing plasmids with no Ubi-tags (D), Ubi-R tags(E), Ubi-R-Δk tags(F) fused to the *N*-terminus of the Leu2 and Ubi-R-Δk tags fused to the *N*-terminus of the Hgy **(G)**. Determination of the loss rate of different plasmids in the rich(H) or selective medium (I).(J) The cell growth of transformants containing different plasmids. Data represent the mean ± SD of two or three biological replicates.

A series of Ubi-tags with different degradation strengths were fused to the *N-*terminus of the Leu2 selection marker. However, no viable transformant carrying the Ubi-R-ek construct was obtained under selective conditions. This suggested that the excessively strong degradation activity of the Ubi-R-ek tag reduced selection marker abundance below a functional threshold, thereby preventing transformant growth under selective conditions. Compared with the untagged plasmid, the fluorescence intensities of YLEP-*UBI*-R-*LEU2* and YLEP-*UBI*-R-Δk-*LEU2* plasmids were 14.7% and 228.1% higher, respectively (Figs. 3D, E, F). Plasmid copy number analysis further showed that YLEP-*UBI*-R-*LEU2* and YLEP-*UBI*-R-Δk-*LEU2* were maintained at an average of approximately 1.6 and 5.0 copies per cell, representing 37.9% and 356.36% increase relative to the untagged plasmids (Figs. 3D, E, F).

To determine whether Ubi-mediated modulation of selection marker abundance is influenced by the type of selective marker, we replaced the marker *LEU2* with the antibiotic marker *HYG*. As shown in Fig. 3F, G, the fluorescence intensity and plasmid copy number between the two plasmids with different marker were similar. Collectively, these findings indicated that Ubi-mediated copy number enhancement was largely independent of marker type.

To further assess plasmid performance, plasmid loss rates were quantified under both selective and non–selective conditions. As shown in Fig. 3H-I, all three plasmids showed comparable loss rates. Although the strain carrying YLEP-*UBI*-R-Δk-*LEU2* plasmids exhibited a modest reduction in biomass accumulation during the early exponential phase, its cell density at the later stage was comparable to that of the other constructs (Fig. 3J). Collectively, these results demonstrated that Ubi-mediated modulation of selection marker abundance could substantially increase plasmid copy number while maintaining genetic stability and without compromising cell growth significantly.

To further enhance plasmid copy number, the truncation of promoter of *LEU2* was introduced in the plasmid YLEP-*UBI*-R-Δk-*LEU2*. The promoter (290 bp) was shortened to 200, 150, and 100 bp. However, these truncations did not result in further improvement of GFP fluorescence intensity (Fig. 4). This indicated that the use of the Ubi–tag is a more direct and effective approach for modulating selection marker abundance than adjusting promoter strength.

**Figure 4:**
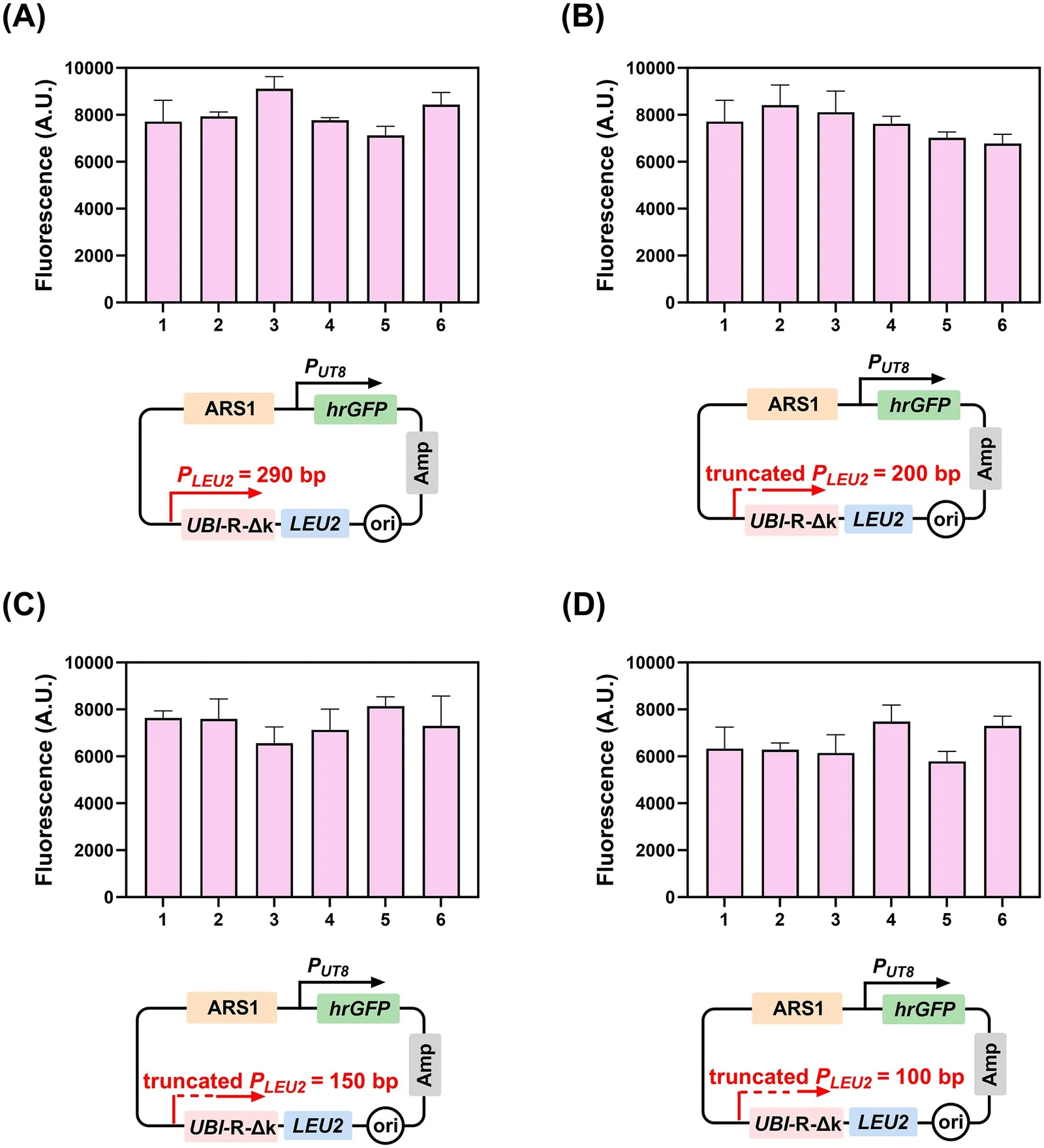
Effect of truncated promoters of *LEU2* on the fluorescence intensity of YLEP-*UBI*-R-Δk-*LEU2* plasmid.

### Application of the optimized plasmid for β-carotene biosynthesis

To evaluate the potential of the engineered plasmid for gene expression, β-carotene biosynthesis pathway was constructed to the engineered plasmid. As shown in Fig. 5A, the genes encoding phytoene synthase/lycopene cyclase (CarRP) and phytoene dehydrogenase (CarB) from *Mucor circinelloides* were expressed in YLEP-ORICEN-*hrGFP*, YLEP–ARS1–*hrGFP* and YLEP-*UBI*-R-Δk-*LEU2* plasmids to generate YLEP-ORICEN-*CarRP*-*CarB*, YLEP–ARS1–*CarRP*-*CarB* and YLEP *UBI*-R-Δk-*CarRP*-*CarB*, respectively. These plasmids were then transformed into *Y. lipolytica* Po1f to generate strains Car1, Car2 and Car3, respectively. Meanwhile, the YLEP-ORICEN plasmid without the gene of *CarRP* and *CarB* was also transformed into Po1f to generate a control strain Car0. As shown in Fig. 5B and C, the strains Car1, Car2 and Car3 produced 6.75, 9.86 and 29.94 mg/L β-carotene, respectively, representing approximately a 46.1% increase for Car2 and a 343.6% increase for Car3 relative to the non–optimized plasmid (Car1). No β–carotene was detected in the control strain Car0. The copy numbers of *CarRP* and *CarB* were significantly higher in Car3 (4.7 copies per cell) than in Car1 (1.1 copies per cell) and Car2 (1.5 copies per cell) (Fig S2).

**Figure 5:**
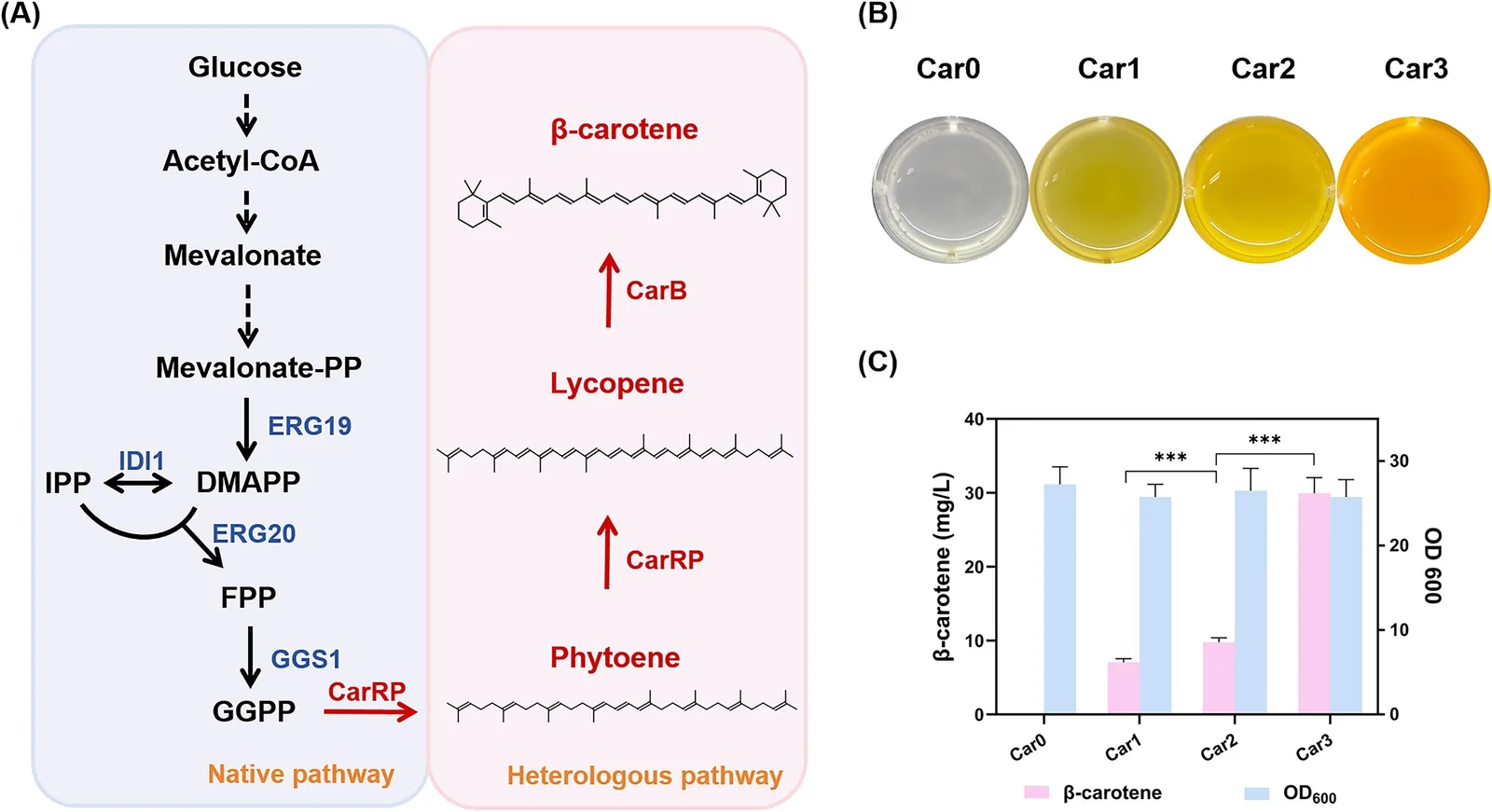
Application of the optimized plasmid for β-carotene biosynthesis. (A) β-Carotene biosynthesis pathway in engineered the *Y. lipolytica*. ERG19, mevalonate diphosphate decarboxylase; IDI1, isopentenyl diphosphate delta-isomerase; ERG20, geranyl/farnesyl diphosphate synthase; GGS1, GGPP synthase; CarRP, bifunctional enzymes phytoene synthase/lycopene cyclase; and CarB, phytoene dehydrogenase.(B) The color change of different strains. Strain Car0 (carrying YLEP-ORICEN), Car1 (carrying YLEP-ORICEN-*CarRP*-*CarB*), Car2 (carrying YLEP-ARS1-*CarRP*-*CarB*) and Car3 (carrying YLEP *UBI*-R-Δk-*CarRP*-*CarB*) were incubated at 30°C and 220 rpm for 72 h and take pictures. **(C)** β-carotene production in different strains transformants. Data represent the mean ± SD of two or three biological replicates. Statistical significance was determined using a two-tailed *t*-test. ***, *P* < 0.001.

These results demonstrated that the engineered episomal plasmid significantly enhanced protein expression capacity in *Y. lipolytica*. Thus, the engineered plasmid represents a promising tool for high level gene expression in non–conventional yeasts.

## Discussion

Currently, the most commonly used ARS elements in episomal plasmids for *Y. lipolytica* are derived from chromosomal DNA and mitochondrial DNA (Table 2). ARS1 and ORI-CEN are commonly used chromosomal ARS elements. ORI-CEN is a truncated version of ARS1 lacking the intervening linker region. Our results indicate that, although the linker region is not essential for plasmid replication and segregation, it significantly influences plasmid stability and gene expression level. It is reported that altering the length or composition of the native linker significantly affect plasmid stability and heterologous protein expression in *Y. lipolytica* without significantly altering the plasmid copy number (Lopez et al. 2021, Li et al. 2026). Although the precise molecular mechanisms remain incompletely understood, there is evidence suggesting that the linker sequence participates in coordinating plasmid segregation, shaping local DNA architecture that influences episomal behavior (Matsuoka et al. 1993).

**Table 2:** Comparison of chromosomal ARS-based and mitochondrial ARS-based episomal plasmids in *Y. lipolytica*.

Feature	Chromosomal ARS (e.g. ARS1)	Mitochondrial ARS (e.g.mtORI)
Origin	Chromosomal DNA	Mitochondrial DNA
Replication control	Cell cycle-dependent	Relatively independent
Copy number	Low	Highly variable
Expression level	High	Medium
Plasmid loss rate	Medium	Low

Currently, the episomal plasmids with the highest copy number in *Y. lipolytica* are constructed based on mitochondrial-derived ARS elements. Owing to distinct replication mechanisms of yeast mitochondrial DNA (e.g. rolling-circle-like processes and relaxed cell cycle regulation), plasmids carrying mitochondrial ARS elements exhibit considerable copy number fluctuations (Chen et al. 2018). It has been shown that episomal plasmids based on the *Y. lipolytica* mtORI achieved a copy number of up to 5 copies per cell after optimizing the selection marker concentration (Cui et al. 2021). In contrast, a copy number of up to 9 copies per cell has been reported using mtORI-based episomal plasmids without optimization of other plasmid elements (Yang et al. 2024). We speculate that higher copy numbers of mtORI plasmid observed in their study may be attributable to differences in strain background and selection marker. In addition, the differences in DNA extraction efficiency, primer design, and other aspects of qPCR assays may also contribute to the variation in reported copy numbers.

Despite the high copy numbers achievable with mtORI, our results indicate that at equal copy numbers, ARS1-based plasmids achieve higher protein expression levels than mtORI-based plasmids. We speculate that this discrepancy may be caused by the fact that autonomous replication sequences influence not only plasmid replication but also transcriptional output. For example, the spacer region within ARS1 has been reported to enhance both mitotic stability and gene transcription (Lopez et al. 2021). Therefore, even when ARS1- and mtORI-based plasmids exhibit similar average copy numbers, their transcriptional efficiency may differ.

To improve the copy number of ARS1-based plasmids, reducing marker abundance using Ubi-tags was performed. Fusion of a Ubi-R tag to the selection marker increased copy number by 80% in *S. cerevisiae* (Chen et al. 2012) and enhanced genomic copy number fourfold in *Y. lipolytica* (Sun et al. 2026). Notably, in this study, incorporation of the Ubi–R–Δk tag resulted in an approximately fourfold increase in plasmid copy number, exceeding the effect of the conventional Ubi–R tag. To our knowledge, this work represents the first application of the Ubi–R–Δk tag for plasmid copy number control, establishing it as a novel and versatile tool for tuning gene dosage in synthetic biology. The engineered plasmid developed herein achieved an average copy number of 5 copies per cell, comparable to that of mtORI–based episomal plasmids. Importantly, this system integrates the high expression capacity of ARS1-based plasmids with the elevated copy number.

In this study, we developed a robust episomal plasmid platform in *Y. lipolytica*. ARS1 was identified as the optimal ARS element for balancing stability and expression. The Ubi–R–Δk tag was implemented to fine–tune selection marker abundance. The engineered plasmid achieved an average copy number of approximately 5 copies per cell, a level comparable to the highest reported in *Y. lipolytica*. Moreover, this plasmid is more suitable for high-level expression of the target gene. To further evaluate the engineered plasmid, we applied it to β–carotene biosynthesis. The results showed that the β–carotene titer was 3.43–fold higher than that of the non–optimized plasmid. This work expands the episomal plasmid toolbox for *Y. lipolytica* and provides a generalizable framework for engineering robust protein expression plasmids in other non–conventional yeasts.

## Supplementary Material

foag054_Supplemental_File

## Data Availability

The original/source data and resources are available from the correspondence author Jin Hou (houjin@sdu.edu.cn). on request.
