# Supplementary material for: Engineering a stable high-copy episomal plasmid in Yarrowia lipolytica
Source: FEMS Yeast Res. 2026 Oct 13;26:foag054. doi: 10.1093/femsyr/foag054 (PMC13685787; doi:10.1093/femsyr/foag054)
Supplement: Supplementary file 1 [file foag054_supplemental_file.docx]

**Supporting data**

**Engineering a stable high-copy episomal plasmid in *Yarrowia lipolytica***

Fengyi Li, Sirui Peng, Huangwei Xu, Yalin Guo, Qingsheng Qi*, and Jin Hou*

^a^Shandong University, State Key Laboratory of Microbial Technology, Binhai Road 72, Qingdao, 266237, P. R. China.

*To whom correspondence should be addressed:

Jin Hou, Email: houjin@sdu.edu.cn.

Qingsheng Qi, Email: qiqingsheng@sdu.edu.cn.

**Figure S1** The plasmid copy number of YLEP-ORICEN-*hrGFP*, YLEP-ARS1-*hrGFP* and YLEP-mtORI-*hrGFP* in selective medium during the loss rate assay under (A) batch cultivation and (B) serial subculturing (three subcultures S1, S2 and S3; 1:100 dilution every 48 h, ~144 h total). Data are presented as means ± SD from three independent biological replicates.

**Figure S2** The copy numbers of CarRP and CarB in different strains. Data represent the mean ± SD of two biological replicates.

**Table S1.** Strains and Plasmids used in this study.

| **Strains/Plasmids** | **Description** | **Sources** |
| --- | --- | --- |
| Po1f | *MatA, leu2-270, ura3-302, xpr2-322, axp-2* | Laboratory stock |
| Car0 | Po1f carrying plasmid YLEP-ORICEN | This study |
| Car1 | Po1f carrying plasmid YLEP-ORICEN-*CarRP-CarB* | This study |
| Car2 | Po1f carrying plasmid YLEP-*UBI*-R-Δk-leu-*CarRP-CarB* | This study |
| YLEP-ORICEN-*hrGFP* | Episomal vector with the ORI-CEN, *LEU2* marker and  *hrGFP* expression cassette | Laboratory stock |
| YLEP-mtORI-  *hrGFP* | Episomal vector with the mtORI, *LEU2* marker and  *hrGFP* expression cassette | This study |
| YLEP-ARS1-  *hrGFP* | Episomal vector with the ARS1, *LEU2* marker and  *hrGFP* expression cassette | This study |
| YLEP-*UBI*-R-  *LEU2* | Episomal vector with the ARS1, *Ubi-R-LEU2* marker and  *hrGFP* expression cassette | This study |
| YLEP-*UBI*-R-ek-  *LEU2* | Episomal vector with the ARS1, *Ubi-R-ek-LEU2* marker and  *hrGFP* expression cassette | This study |
| YLEP-*UBI*-R-Δk-*LEU2* | Episomal vector with the ARS1, *Ubi-R-Δk-LEU2* marker and  *hrGFP* expression cassette | This study |
| YLEP-*UBI*-R-Δk-*HYG* | Episomal vector with the ARS1, *Ubi-R-Δk-HYG* marker and  *hrGFP* expression cassette | This study |
| YLEP-leu200 | YLEP-Ubi-R-Δk-leu-hrGFP plasmid containing *LEU2* with 200 bp truncated promoter | This study |
| YLEP-leu150 | YLEP-Ubi-R-Δk-leu-hrGFP plasmid containing *LEU2* with 150 bp truncated promoter | This study |
| YLEP-leu100 | YLEP-Ubi-R-Δk-leu-hrGFP plasmid containing *LEU2* with 100 bp truncated promoter | This study |
| YLEP-ORICEN-*CarRP-CarB* | Episomal vector with the ORI-CEN, *LEU2* marker, *CarRP* and *CarB* expression cassette | This study |
| YLEP-ARS1 -*CarRP-CarB* | Episomal vector with the ARS1, *LEU2* marker *CarRP* and *CarB* expression cassette | This study |
| YLEP-*UBI*-R-Δk-leu-*CarRP-CarB* | Episomal vector with the ARS1, Ubi-R-Δk-*LEU2* marker, *CarRP* and *CarB* expression cassette | This study |
| YLEP-ORICEN | Episomal vector with the ORI-CEN and *LEU2* marker | This study |

**Table S2.** Primers used in this study.

| **Name** | **Sequence (5′→3′)** | **Use** |
| --- | --- | --- |
| ARS1-S | GCTTTAATTTGCCTCATTTACAATTTCATCTTTCATCA | Amplification of ARS1 |
| ARS1-A | ACGACGGAATTCCATACTACTGTATATTCAAGCAAGT |  |
| mtORI-S | GCTTTAATTTGCAACCTGTACCCTTAAGCATT | Amplification of mtORI |
| mtORI-A | CGACGGAATTCGGAACTAAACTAACAACATTAGCT |  |
| YLEP-S | GAATTCCGTCGTCGCCTGA | Amplification of vector without ORI-CEN |
| YLEP-A | GCAAATTAAAGCCTTCGAGC |  |
| Ubi-R-S | CATACAACCACACACATCCACAATGCAAATCTTTGTCAAGACC | Amplification of *UBI*-R |
| Ubi-R-A | GTTTCGGGTTCCATTCGACCTCCTCGGAGTCGCAG |  |
| Ubi-R-ek-S | CATACAACCACACACATCCACAATGCAAATCTTTGTCAAGACC | Amplification of *UBI*-R-ek |
| Ubi-R-ek-A | CTTAGTTTCGGGTTCCATGGATCGCTGCATCAGAGAG |  |
| Ubi-R-Δk-S | CATACAACCACACACATCCACAATGCAAATCTTTGTCAAGACC | Amplification of *UBI*-R-Δk |
| Ubi-R-Δk-A | TAGTTTCGGGTTCCATGGTGTTTGGAGCCAGAGTGG |  |
| YLEP-Ubi-S | ATGGAACCCGAAACTAAGAAG | Amplification of YLEP-ARS1-*hrGFP* |
| YLEP-Ubi-A | TGTGGATGTGTGTGGTTGTATG |  |
| Hyg-s | CCAAACACCATGAAAAAGCCTGAACTCACC | Amplification of *HYG* |
| Hyg-a | CGTAGAAACGACCTATTCCTTTGCCCTCGGAC |  |
| YLEP-Hyg-S | CGTAGAAACGACCTATTCCTTTGCCCTCGGAC | Amplification of YLEP-*UBI*-R-Δk-*LEU2* without *LEU2* |
| YLEP-Hyg-A | CAGGCTTTTTCATGGTGTTTGGAGCCAGAGTGG |  |
| Leu200-S | AGTAGTATGGTACGTTCGATAGCGCTATCGG | Construction of YLEP-leu200 |
| Leu200-A | ATCGAACGTACCATACTACTGTATATTCAAGCAAGT |  |
| Leu150-S | ACAGTAGTATGTTGGGTCCCTAGCCGATAC | Construction of YLEP-leu150 |
| Leu150-A | AGGGACCCAACATACTACTGTATATTCAAGCAAGT |  |
| Leu100-S | ACAGTAGTATGGGTTTAGTCTTCCACATAGCAC | Construction of YLEP-leu100 |
| Leu100-A | GAAGACTAAACCCATACTACTGTATATTCAAGCAAGT |  |
| CarRP+B-S | CAAAGGTTCGAAATGTCCAAGAAGCACATTGTG | Amplification of *CarRP* and *CarB* |
| CarRP+B-A | GCCTTCGAGCAGTGTGGGAGGTTAAGAG |  |
| YLEP-Z-S | CTCCCACACTGCTCGAAGGCTTTAATTTGC | Amplification of YLEP-*UBI*-R-Δk-*LEU2,* YLEP-ARS1 -*hrGFP* or YLEP-ORICEN-*hrGFP* without *hrGFP* |
| YLEP-Z-A | GCTTCTTGGACATTTCGAACCTTTGAATGATTCTTATACTC |  |

**Table S3.** qPCR primers used in this study.

| **Name** | **Sequence (5′→3′)** | **Use** |
| --- | --- | --- |
| GAPDH-S | CGGACGAATCGGACGAATTGTGA | Amplification of *GAPDH* |
| GAPDH-A | CAGCGTACTCGGTGTCGATGAAG |  |
| hrGFP-S | GACGGCCCCGTGATGAAGAAG | Amplification of *GFP* |
| hrGFP-A | TCTACGAAGCCGCCGTCCTCC |  |
| CarRP-S | CGCTTGGGACAACTACATTGTC | Amplification of *CarRP* |
| CarRP-A | CCATCGCATGACCAGGTTAG |  |

**Table S4.** Sequences of heterologous proteins used in this study.

| **Name** | **Sequence** | **Genbank No.** |
| --- | --- | --- |
| CarRP | MLLTYMEVHLYYTLPVLGVLSWLSRPYYTATDALKFKFLTLVAFTTASAWDNYIVYHKAWSYCPTCVTAVIGYVPLEEYMFFIIMTLLTVAFTNLVMRWHLHSFFIRPETPVMQSVLVRLVPITALLITAYKAWHLAVPGKPLFYGSCILWYACPVLALLWFGAGEYMMRRPLAVLVSIALPTLFLCWVDVVAIGAGTWDISLATSTGKFVVPHLPVEEFMFFALINTVLVFGTCAIDRTMAILHLFKNKSPYQRPYQHSKSFLHQILEMTWAFCLPDQVLHSDTFHDLSVSWDILRKASKSFYTASAVFPGDVRQELGVLYAFCRATDDLCDNEQVPVQTRKEQLILTHQFVSDLFGQKTSAPTAIDWDFYNDQLPASCISAFKSFTRLRHVLEAGAIKELLDGYKWDLERRSIRDQEDLRYYSACVASSVGEMCTRIILAHADKPASRQQTQWIIQRAREMGLVLQYTNIARDIVTDSEELGRCYLPQDWLTEKEVALIQGGLAREIGEERLLSLSHRLIYQADELMVVANKGIDKLPSHCQGGVRAACNVYASIGTKLKSYKHHYPSRAHVGNSKRVEIALLSVYNLYTAPIATSSTTHCRQGKMRNLNTI | KAL9541990.1 |
| CarB | MSKKHIVIIGAGVGGTATAARLAREGFKVTVVEKNDFGGGRCSLIHHQGHRFDQGPSLYLMPKYFEDAFADLDERIQDHLELLRCDNNYKVHFDDGESIQLSSDLTRMKAELDRVEGPLGFGRFLDFMKETHIHYESGTLIALKKNFESIWDLIRIKYAPEIFRLHLFGKIYDRASKYFKTKKMRMAFTFQTMYMGMSPYDAPAVYSLLQYTEFAEGIWYPRGGFNMVVQKLEAIAKQKYDAEFIYNAPVAKINTDDATKQVTGVTLENGHIIDADAVVCNADLVYAYHNLLPPCRWTQNTLASKKLTSSSISFYWSMSTKVPQLDVHNIFLAEAYQESFDEIFKDFGLPSEASFYVNVPSRIDPSAAPDGKDSVIVLVPIGHMKSKTGDASTENYPAMVDKARKMVLAVIERRLGMSNFADLIEHEQVNDPAVWQSKFNLWRGSILGLSHDVLQVLWFRPSTKDSTGRYDNLFFVGASTHPGTGVPIVLAGSKLTSDQVVKSFGKTPKPRKIEMENTQAPLEEPDAESTFPVWFWLRAAFWVMFMFFYFFPQSNGQTPASFINNLLPEVFRVHNSNVI | KAL9541991.1 |
| Ubi-R | MQIFVKTLTGKTITLEVESSDTIDNVKSKIQDKEGIPPDQQRLIFAGKQLEDGRTLSDYNIQKESTLHLVLRLRGGR | XP_018226571.1 |
| Ubi-R-Δk | MQIFVKTLTGKTITLEVESSDTIDNVKSKIQDKEGIPPDQQRLIFAGKQLEDGRTLSDYNIQKESTLHLVLRLRGGRHGSGAWLLPVSLVKRKTTLAPNT | AFQ55252.1 |
| Ubi-R-ek | MQIFVKTLTGKTITLEVESSDTIDNVKSKIQDKEGIPPDQQRLIFAGKQLEDGRTLSDYNIQKESTLHLVLRLRGGRHGSGAWLLPVSLVKRKTTLAPNTASPRALADSLMQRS | AFQ55252.1 |
